# Serum IL-6 and IL-10 in Early Childhood Caries: A Sibling-Controlled Study in Children Aged 2.5–6 Years

**DOI:** 10.3390/diagnostics16142210

**Published:** 2026-07-15

**Authors:** Ștefania Alice Petrache, Ionela Teodora Dascălu, Lidia Boldeanu, Oana Andreea Diaconu, Ana Maria Rîcă, Lelia Mihaela Gheorghiță, Mihaela Jana Țuculină, Constantin Dăguci, Paula Perlea, Mădălina Olteanu

**Affiliations:** 1 Doctoral School, University of Medicine and Pharmacy of Craiova, 200349 Craiova, Romania; stefaniapetrache90@gmail.com; 2 Department of Orthodontics, Faculty of Dental Medicine, University of Medicine and Pharmacy of Craiova, 200349 Craiova, Romania; marceldascalu@yahoo.com (I.T.D.); m03olteanu@gmail.com (M.O.); 3 Department of Microbiology, Faculty of Medicine, University of Medicine and Pharmacy of Craiova, 200349 Craiova, Romania; 4 Department of Endodontics, Faculty of Dental Medicine, University of Medicine and Pharmacy of Craiova, 200349 Craiova, Romania; oanamihailescu76@yahoo.com (O.A.D.); r_ana_maria22@yahoo.com (A.M.R.); leliagheorghita@yahoo.com (L.M.G.); 5 Department of Oro-Dental Prevention, Faculty of Dental Medicine, University of Medicine and Pharmacy of Craiova, 200349 Craiova, Romania; dagucicristi@yahoo.com; 6 Department of Endodontics, Faculty of Dental Medicine, University of Medicine and Pharmacy Carol Davila of Bucharest, 050474 Bucharest, Romania; paula.perlea@gmail.com

**Keywords:** early childhood caries, sibling controls, IL-6, IL-10, inflammatory biomarkers, oral hygiene, plaque index, gingival index, hematologic inflammatory indices, cytokines

## Abstract

**Background**: Early childhood caries (ECC) is a biofilm-mediated and sugar-driven disease associated with local and systemic inflammatory responses. This study evaluated serum Interleukin-6 (IL-6) and IL-10 concentrations, hematologic inflammatory indices, and oral inflammatory parameters in children with ECC compared with caries-free sibling controls. **Methods**: This sibling-controlled case–control study included 155 children aged 2.5–6 years, comprising 120 children with active ECC and 35 caries-free siblings. Serum IL-6 and IL-10 concentrations were measured using enzyme-linked immunosorbent assay (ELISA). Complete blood count parameters and derived inflammatory indices, including neutrophil-to-lymphocyte ratio (NLR), lymphocyte-to-monocyte ratio (LMR), systemic immune-inflammation index (SII), systemic inflammation response index (SIRI), aggregate index of systemic inflammation (AISI), cumulative inflammatory index (IIC), and mean corpuscular volume-to-lymphocyte ratio (MCVL), were calculated. Plaque Index (PI) and Gingival Index (GI) were recorded in children with ECC. Group comparisons, correlation analyses, ROC analysis, and multivariable logistic regression were performed. **Results**: Children with ECC exhibited significantly higher serum IL-6 and IL-10 concentrations than sibling controls. MCVL values were significantly lower in the ECC group, whereas several inflammatory indices indicated an increased systemic inflammatory burden. Stratification by PI tertiles showed progressively higher values for WBC, NEU, NLR, SII, SIRI, AISI, and IIC as plaque accumulation increased (all *p* < 0.05). PI was strongly correlated with GI (r = 0.811, *p* < 0.001) and moderately correlated with NLR (r = 0.536), SII (r = 0.536), SIRI (r = 0.654), AISI (r = 0.648), and IIC (r = 0.546) (all *p* < 0.01). Serum IL-6 and IL-10 were strongly correlated (r = 0.804, *p* < 0.001) but not with PI or GI. ROC analysis demonstrated moderate discriminatory performance for IL-6 (AUC = 0.701) and IL-10 (AUC = 0.696). Age- and sex-adjusted multivariable logistic regression demonstrated that elevated IL-6 concentrations (adjusted OR = 1.36; 95% CI, 1.10–1.68; *p* = 0.005) remained independently associated with ECC, whereas increasing MCVL values (adjusted OR = 0.92; 95% CI, 0.86–0.98; *p* = 0.010) were associated with reduced odds of ECC. **Conclusions**: ECC is associated with systemic cytokine alterations and increased hematologic inflammatory burden. While IL-6 and IL-10 distinguish children with ECC from sibling controls, plaque accumulation severity is better reflected by composite hematologic inflammatory indices than by isolated cytokine concentrations. IL-6 and MCVL may be promising adjunctive biomarkers for ECC.

## 1. Introduction

Early childhood caries (ECC) is a multifactorial, biofilm-mediated, and sugar-driven disease characterized by demineralization of primary teeth. Its development is influenced by dietary habits, oral hygiene, enamel susceptibility, microbial biofilm composition, fluoride exposure, and socioeconomic factors. Although dental caries itself is primarily a hard-tissue demineralization process, advanced lesions and associated oral conditions may coexist with local tissue responses and measurable systemic biological changes [1,2]. Recent evidence suggests that untreated dental caries may negatively affect nutrition, growth, sleep quality, school attendance, and psychosocial well-being in pediatric populations [3]. Current evidence recognizes ECC as a dysbiosis-driven, biofilm-mediated, and sugar-dependent disease resulting from a prolonged ecological imbalance within the oral microbiome, ultimately leading to progressive demineralization of primary teeth [4,5].

Inflammatory cytokines have gained increasing attention as potential biomarkers reflecting disease activity and the host immune response in children with ECC. Interleukin 6 (IL-6) and IL-10 may provide complementary information regarding the host biological response in children with early childhood caries [6]. IL-6 is a pleiotropic pro-inflammatory cytokine involved in leukocyte recruitment, acute-phase reactions, B-cell differentiation, and osteoclast activation [7]. Conversely, IL-10 exerts predominantly anti-inflammatory and immunomodulatory effects by suppressing excessive cytokine production and limiting tissue damage [8].

Several recent studies have demonstrated altered cytokine profiles in children with active dental caries. Elevated salivary IL-6 levels have been associated with increased cariogenic activity and oral inflammatory burden, while IL-10 may reflect compensatory regulatory responses to chronic bacterial stimulation [9,10,11]. Systematic reviews and meta-analyses further support the role of inflammatory mediators as potential diagnostic biomarkers in ECC [12]. Nevertheless, most studies currently available primarily focus on salivary biomarkers, whereas relatively limited data are available on systemic inflammatory responses reflected in serum cytokine concentrations.

Accumulation of dental plaque and inadequate oral hygiene are considered key contributors to oral dysbiosis and gingival inflammation. Clinical indices such as the Plaque Index (PI) and Gingival Index (GI) are widely used to evaluate plaque accumulation, gingival inflammatory changes, and oral hygiene status [13,14,15]. Recent investigations demonstrated significant associations between poor oral hygiene indices and increased inflammatory cytokine expression in saliva and gingival crevicular fluid in pediatric populations [16,17]. However, studies simultaneously evaluating serum IL-6 and IL-10 concentrations together with multiple oral hygiene and gingival indices in children with dental caries remain scarce.

Furthermore, sibling-based control designs are rarely used in pediatric oral inflammatory research despite their methodological advantages. The use of sibling controls may reduce confounding from shared environmental exposures, dietary habits, socioeconomic background, and genetic susceptibility, thereby enabling a more reliable assessment of the association between oral inflammatory burden and systemic immune response [18,19,20,21].

Therefore, the primary aim of the present study was to compare serum IL-6 and IL-10 concentrations between children aged 2.5–6 years with ECC and caries-free sibling controls. Secondary aims were to evaluate selected hematologic parameters and derived inflammatory indices in relation to caries status and to explore whether IL-6 and IL-10 are associated with clinical oral parameters recorded in this cohort.

## 2. Materials and Methods

### 2.1. Study Design and Participants

This case–control study included 155 pediatric participants aged 2.5–6 years, recruited from the Department of Pediatric Dentistry between October 2024 and March 2026. The study group consisted of 120 children diagnosed with active dental caries, while the control group included 35 caries-free siblings of the affected patients. The sibling-control design was selected to minimize potential confounding factors related to shared environmental exposures, dietary habits, socioeconomic backgrounds, and genetic susceptibility. The control group comprised caries-free siblings who were present only among a subset of the children included in the ECC group, as not every child with ECC had an eligible caries-free sibling who met the inclusion criteria. Consequently, the study was designed as a case–control study including a sibling control subgroup rather than as a complete one-to-one matched sibling-pair study.

All participants underwent a clinical oral examination and had blood samples collected for assessment of inflammatory biomarkers. Demographic data, oral hygiene indices, gingival status, and serum cytokine levels were recorded for all enrolled subjects.

The study protocol was conducted in accordance with the Declaration of Helsinki and was approved by the Ethics Committee of the University of Medicine and Pharmacy of Craiova, which served as the study’s primary ethics authority (no. 123/15 June 2023). Written informed consent was obtained from the parents or legal guardians of all participants prior to enrollment.

The present cohort was recruited independently from the previously published sibling-controlled study and includes no participants from that study [22]. The current analysis addresses a distinct research question by focusing on serum IL-6 and IL-10 and their associations with PI and GI in children aged 2.5–6 years. The previous article is cited for transparency and to clarify that the present manuscript reports an independent dataset.

### 2.2. Patient Selection

#### 2.2.1. Inclusion Criteria

Caries Group—children were included in the caries group if they fulfilled the following criteria:

- age between 2.5 and 6 years [5];
- presence of active dental caries diagnosed during clinical examination;
- absence of systemic inflammatory or autoimmune diseases;
- absence of acute infectious disease at the time of examination;
- no antibiotic or anti-inflammatory treatment within the previous 30 days;
- availability of complete clinical and laboratory data;
- signed informed consent from parents or legal guardians.

Control Group—children were included in the control group if they met the following criteria:

- Sibling of a participant from the caries group;
- Absence of clinically detectable active dental caries;
- Absence of systemic inflammatory or autoimmune diseases;
- No acute infection at the time of examination;
- No antibiotic or anti-inflammatory therapy within the previous 30 days;
- Signed informed consent from parents or legal guardians.

#### 2.2.2. Exclusion Criteria

Participants were excluded from the study in the presence of any of the following conditions:

- Systemic inflammatory, autoimmune, metabolic, or immunological disorders;
- Current infectious diseases or fever;
- Oral mucosal lesions, gingival conditions, or other oral findings that could interfere with reliable clinical assessment;
- Use of antibiotics, corticosteroids, or anti-inflammatory medication within 30 days prior to enrollment;
- Incomplete clinical or laboratory data;
- Children with oral conditions or appliances that prevented reliable clinical examination;
- Refusal to participate in the study.

### 2.3. Clinical Oral Examination

Active ECC was defined as the presence of at least one untreated cavitated carious lesion in a primary tooth. Caries status was recorded dichotomously as present or absent based on clinical examination.

The study was designed to compare children with clinically diagnosed active ECC and caries-free sibling controls. Accordingly, participants were classified by the presence or absence of untreated cavitated lesions rather than by quantitative caries severity indices.

Clinical examinations were performed by a single calibrated examiner under standardized conditions using dental mirrors and periodontal probes.

Oral hygiene and gingival status were assessed using the following validated clinical indices:

- Plaque Index (PI)—In order to estimate the presence of plaque at the gingival margin of the teeth, the plaque index (PI), was used. According to the recording protocols for this index (the criteria proposed by Silness and Löe), each of the six gingival tooth surfaces was examined with a probe, and a score ranging from 0 to 3 was assigned to each. The mean of the values recorded for each tooth determines the patient’s score [13,23].
  – Scoring Criteria: Each surface is given a score based on the amount of plaque:
    ➢ Score 0: No plaque.
    ➢ Score 1: A film of plaque that is not visible to the naked eye but can be seen with a disclosing solution or a probe.
    ➢ Score 2: Moderate accumulation of soft deposits visible without any aids.
    ➢ Score 3: Abundant soft matter within the gingival pocket or on the tooth and gingival margin.
  – Interpretation for scores:
    ➢ 0: Excellent hygiene.
    ➢ 0.1–0.9: Good hygiene.
    ➢ 1.0–1.9: Fair hygiene.
    ➢ 2.0–3.0: Poor hygiene.
- Gingival Index (GI)—GI scores were assessed according to Löe and Silness criteria; they were recorded using clinical inspection and probing; the score was calculated with the ratio of the mean score of the teeth/number of teeth examined [13,15]. The occurrence of gingival inflammation at four surfaces of each tooth was assessed using the criteria of the gingival index system, as follows:
  – <0.1: no inflammation;
  – 0.1–1: mild inflammation, slight change in color, slight edema, no bleeding on probing;
  – 1.1–2: moderate inflammation, redness, edema and glazing, bleeding on probing;
  – 2.2–3: severe inflammation, marked redness and edema, ulceration, tendency for spontaneous bleeding.

### 2.4. Laboratory Investigations

#### Sample Collection

Blood samples were collected during routine clinical laboratory investigations performed as part of the participants’ standard medical evaluation. No additional venipuncture was performed solely for research purposes. Following written informed consent from the children’s legal guardians, serum samples obtained during routine blood collection were used for the immunological analyses performed in the present study. To minimize unnecessary invasive procedures in this pediatric population, only children for whom routine blood sampling was clinically indicated and successfully completed were eligible for inclusion. Consequently, although they fulfilled the clinical inclusion criteria, 45 otherwise eligible children were not enrolled because biological samples could not be obtained during routine clinical evaluation. Blood collection was performed by experienced pediatric healthcare personnel in accordance with our institution’s routine procedures. The dental examination and blood sampling were conducted independently as part of the participants’ routine clinical assessment, and study participation did not require any additional invasive procedures beyond standard clinical care. No clinically relevant difficulties related to participant cooperation were observed that interfered with the dental examination, blood collection, or subsequent laboratory analyses. Although every effort was made to ensure uniform sample handling, fasting status, exact blood collection time, and the interval between blood collection and sample processing were not prospectively standardized or systematically recorded for all participants.

Two blood samples were collected from each participant and processed as follows:

- Serum samples—Two additive-free tubes were collected, about 5 mL of venous blood from each patient. Following standard procedures, samples were allowed to clot, then centrifuged at 3000× *g* for 10 min within 4 h of collection using a Hermle centrifuge (Hermle AG, Gosheim, Baden-Württemberg, Germany). Serum from one tube was aliquoted into labeled vials, sealed tightly, and stored at −20 °C to −80 °C, avoiding freeze–thaw cycles. Before analysis, frozen serum was passively thawed to room temperature. These aliquots were used for immunological tests, while serum from the second tube was used for biochemical analyses.
- Hematologic analyses—Peripheral venous blood collected in EDTA tubes was used for a complete blood count (CBC) analysis using the MINDRAY BC-6800 hematology analyzer (Mindray, Shenzhen, China). The following hematologic parameters were recorded: hemoglobin (Hb), white blood cells/leukocytes (WBC), neutrophils (NEU), lymphocytes (LYM), monocytes (MON), platelets (PLT), hematocrit (Ht), mean corpuscular volume (MCV), and red cell distribution width (RDW). Inflammation indices such as NLR, LMR, PLR, AISI, SII, SIRI, dNLR, NMR, MCVL, and IIC were calculated from these data:
  – NLR = neutrophil-to-lymphocyte ratio;
  – LMR = lymphocyte-to-monocyte ratio;
  – PLR = platelet-to-lymphocyte ratio;
  – dNLR = derived neutrophil-to-lymphocyte ratio;
  – NMR = neutrophil-to-monocyte ratio;
  – AISI = (neutrophils × monocytes × platelets)/lymphocytes;
  – SII = (neutrophils × platelets)/lymphocytes;
  – SIRI = (neutrophils × monocytes)/lymphocytes;
  – MCVL = mean corpuscular volume to lymphocyte ratio;
  – IIC = (mean corpuscular volume × width of erythrocyte distribution × neutrophils)/(lymphocytes × 1000) [22,24,25,26,27].

Blood samples were collected and processed according to routine standardized procedures of the institutional clinical laboratory. Although every effort was made to ensure uniform sample handling, fasting status, exact blood collection time, and the interval between blood collection and sample processing were not prospectively standardized or systematically recorded for all participants.

### 2.5. Immunological Assessment

Serum levels of IL-6 and IL-10 were determined using commercial ELISA kits from Elabscience (Houston, TX, USA) following the manufacturer’s instructions at the Immunology Laboratory of the University of Medicine and Pharmacy of Craiova.

- Human IL-6 (Interleukin 6) ELISA Kit [Cat. No.: E-EL-H6156; product Link: https://www.elabscience.com/p/human-il-6-interleukin-6-elisa-kit--e-el-h6156 (accessed on 10 April 2026); Sensitivity 0.94 pg/mL; Detection Range 1.56–100 pg/mL; Specificity: Specific for Human IL-6. No significant cross-reactivity or interference with related analogs was observed; Repeatability: Coefficient of variation is <10%; https://789.bio/ea/q9yn1O, (accessed on 10 April 2026)].
- Human IL-10 (Interleukin 10) ELISA Kit [Cat. No.: E-EL-H6154; product Link: https://www.elabscience.com/p/human-il-10-interleukin-10-elisa-kit--e-el-h6154 (accessed on 10 April 2026); Sensitivity 0.94 pg/mL; Detection Range 1.56–100 pg/mL; Specificity: Specific for Human IL-10. No significant cross-reactivity or interference with related analogs was observed; Repeatability: Coefficient of variation is <10%; https://789.bio/ea/affrrD, (accessed on 10 April 2026)].

Each serum sample was analyzed once using the same commercially available ELISA kit and a standardized laboratory protocol. Because of the available sample volume and study resources, duplicate measurements were not performed. Although intra- and inter-assay coefficients of variation were not independently assessed in this study, the analytical performance characteristics of the assays were based on the manufacturer’s validation data.

Test principle—This assay is based on the sandwich ELISA technique. Human IL-6/IL-10 present in the sample is specifically bound by an immobilized capture antibody and subsequently recognized by a biotinylated detection antibody, forming a sandwich immune complex. This complex is further associated with an avidin–horseradish peroxidase (HRP) conjugate, enabling enzymatic signal amplification. Upon reaction with a chromogenic substrate, the HRP enzyme catalyzes the development of a colored product. The resulting signal intensity, measured spectrophotometrically at 450 ± 2 nm, is directly proportional to the concentration of Human IL-6/IL-10 in the sample. Quantitative determination is achieved by comparison with a standard curve generated from known concentrations.

### 2.6. Statistical Analysis

Using Microsoft Excel, we organized and handled patient data from medical records. For data analysis, we utilized GraphPad Prism 11.0.2 (92) (GraphPad Software, LLC, San Diego, CA, USA).

Continuous variables were tested for normality using the Shapiro–Wilk test and are presented as mean ± standard deviation (SD) for normally distributed data or median and interquartile range (IQR) for non-normally distributed variables. Categorical variables are presented as absolute numbers and percentages.

Comparisons between children with ECC and caries-free sibling controls were performed using the independent-samples *t*-test for normally distributed variables and the Mann–Whitney U test for non-normally distributed variables. Differences in categorical variables were evaluated using the chi-square test or Fisher’s exact test, as appropriate. Because the caries-free sibling controls corresponded to only a subset of the children with ECC, the study did not constitute a complete one-to-one matched sibling-pair design. Therefore, statistical comparisons between the ECC and control groups were appropriately performed using independent-samples tests rather than paired tests.

To investigate the association between plaque accumulation severity and systemic inflammatory alterations, children with ECC were stratified into tertiles according to the PI score. Comparisons among PI tertiles were performed using one-way analysis of variance (ANOVA) with Tukey’s post hoc test for normally distributed variables or the Kruskal–Wallis test followed by Dunn’s multiple-comparison test for non-normally distributed variables.

Correlations between clinical oral inflammatory parameters, serum cytokines, and hematologic inflammatory indices were assessed using Spearman’s rank correlation coefficient (r). Correlation strength was interpreted as weak (|r| < 0.30), moderate (|r| = 0.30–0.59), or strong (|r| ≥ 0.60). Correlation matrices were visualized as heatmaps. Given the exploratory nature of the study and the limited sample size, no formal correction for multiple comparisons was applied. Therefore, results with *p*-values close to the statistical significance threshold were interpreted cautiously.

Receiver operating characteristic (ROC) curve analysis was performed to evaluate the ability of serum IL-6 and IL-10 concentrations to discriminate between ECC patients and sibling controls. Diagnostic performance was assessed using the area under the ROC curve (AUC), and optimal cut-off values were determined according to the Youden index. Sensitivity and specificity corresponding to the optimal cut-off values were subsequently calculated.

To identify variables independently associated with ECC while accounting for potential confounding factors, multivariable logistic regression analysis was performed. Because a significant age difference was observed between the ECC and control groups, age and sex were included as adjustment variables in the regression model. Serum IL-6 and IL-10 concentrations, together with PLR and the MCVL. PLR and MCVL were selected as candidate predictors. PLR and MCVL were selected as representative hematologic inflammatory markers to minimize multicollinearity among the highly correlated composite inflammatory indices (NLR, SII, SIRI, AISI, and IIC) while maintaining a parsimonious multivariable model. Results are presented as odds ratios (ORs) with corresponding 95% confidence intervals (95% CIs).

All statistical tests were two-tailed, and a *p*-value < 0.05 was considered statistically significant.

## 3. Results

### 3.1. Baseline Characteristics of the ECC and Sibling Controls

A total of 155 pediatric participants were included in the study, comprising 120 children with ECC and 35 sibling controls. Statistically significant differences were observed in the age distribution between the ECC and sibling controls, with median ages of 57 months and 67 months, respectively (IQR: 47–64 vs. 65–69 months; *p* < 0.0001). The caries group included 62 males and 58 females, whereas the control group included 13 males and 22 females.

Regarding hematological parameters, hemoglobin and hematocrit values were comparable between children with ECC and sibling controls (Hb: 13.13 ± 1.11 g/dL vs. 12.84 ± 0.93 g/dL, *p* = 0.118; Ht: 39.30 ± 3.33% vs. 38.56 ± 2.27%, *p* = 0.134). However, children with ECC presented significantly higher inflammatory cellular markers compared with controls, including WBC (7.38 [5.84–8.97] vs. 6.38 [5.14–6.88] × 10^3^/μL, *p* < 0.001), NEU (2.91 [2.20–4.53] vs. 2.35 [1.88–3.20] × 10^3^/μL, *p* = 0.044), and LYM count (2.94 [2.55–3.46] vs. 2.44 [2.15–3.14] × 10^3^/μL, *p* = 0.020). Consistent with these findings, erythrocyte sedimentation rate (ESR) was also significantly higher in children with ECC than in sibling controls (9.00 [6.00–10.00] vs. 6.00 [5.00–8.00] mm/h, *p* = 0.005).

**Figure 1:**
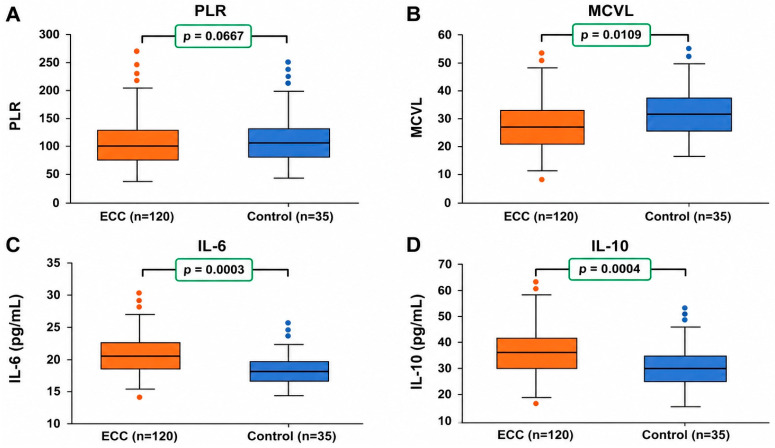
Comparative Boxplot Analysis of PLR, MCVL, IL-6, and IL-10 Between Children with Dental Caries and Sibling Controls. Boxplots illustrating differences in hematologic and inflammatory biomarkers between children with dental caries (*n* = 120) and sibling controls (*n* = 35). Panel (**A**) presents the platelet-to-lymphocyte ratio (PLR); panel (**B**) presents the mean corpuscular volume-to-lymphocyte ratio (MCVL); panel (**C**) presents the serum Interleukin-6 (IL-6) concentration; and panel (**D**) presents the serum Interleukin-10 (IL-10) concentration. Boxes represent the interquartile range (IQR); the horizontal line within each box indicates the median; whiskers correspond to 1.5 × IQR; and dots represent outliers. Statistical comparisons between groups were performed using the Mann–Whitney U test. Significant differences were observed for MCVL (*p* = 0.0109), IL-6 (*p* = 0.00031), and IL-10 (*p* = 0.00042), while PLR showed a borderline trend toward significance (*p* = 0.0667).

Among the hematologic inflammatory indices, most conventional composite inflammatory markers did not differ significantly between ECC patients and sibling controls. Specifically, no significant differences were observed for NLR (*p* = 0.350), LMR (*p* = 0.834), NMR (*p* = 0.183), dNLR (*p* = 0.073), AISI (*p* = 0.123), SII (*p* = 0.396), SIRI (*p* = 0.105), or IIC (*p* = 0.419). PLR demonstrated a borderline difference between groups (99.17 [80.09–125.75] vs. 128.84 [89.92–132.48], *p* = 0.067). Conversely, MCVL values were significantly lower in children with ECC than in sibling controls (28.51 [22.32–32.48] vs. 34.10 [26.51–38.62]; *p* = 0.011) (Figure 1).

Regarding inflammatory biomarkers, serum IL-6 concentrations were significantly higher in children with ECC than in controls (20.70 [18.77–22.15] vs. 18.29 [17.17–19.54] pg/mL, *p* = 0.0003). Similarly, serum IL-10 levels were significantly higher in the ECC group (36.58 [30.49–41.33] vs. 30.29 [25.78–35.63] pg/mL, *p* = 0.0004).

Table 1 summarizes the comparative analysis between ECC and sibling controls.

### 3.2. Sex-Based Comparative Analysis in the ECC Group

Sex-based analysis within the caries group revealed no statistically significant differences regarding age distribution between females and males (60.50 (47.50–65.00) vs. 55.50 (47.00–63.25) months, *p* = 0.365). Hb and Ht values were also comparable between sexes (Hb: 12.65 ± 1.06 g/dL vs. 12.77 ± 1.15 g/dL, *p* = 0.548; Ht: 38.20 ± 2.54% vs. 38.51 ± 2.89%, *p* = 0.536).

No statistically significant sex-related differences were identified for WBC (7.24 [5.80–8.92] vs. 7.41 [5.91–9.01] × 10^3^/μL, *p* = 0.864), NEU (2.88 [2.14–4.41] vs. 2.95 [2.28–4.61] × 10^3^/μL, *p* = 0.771), LYM (2.99 [2.59–3.52] vs. 2.91 [2.50–3.44] × 10^3^/μL, *p* = 0.618), PLT count (298.00 [248.00–360.00] vs. 291.00 [244.00–349.00] × 10^3^/μL, *p* = 0.681), or ESR (9.00 [6.00–14.00] vs. 9.00 [6.00–13.00] mm/h, *p* = 0.941). Similarly, hematologic inflammatory indices, including NLR, PLR, SII, SIRI, AISI, and IIC, showed no statistically significant differences between female and male participants (all *p* > 0.05).

Regarding inflammatory biomarkers, serum IL-6 concentrations were comparable between females and males (22.81 [17.66–28.95] vs. 22.03 [17.11–28.02] pg/mL, *p* = 0.679). However, female participants exhibited significantly higher serum IL-10 levels compared with males (42.11 [34.25–52.36] vs. 39.80 [32.90–49.64] pg/mL, *p* = 0.048). Table 2 summarizes the comparative analysis between female and male participants within the caries group.

### 3.3. PI Stratification Analysis

To further investigate the relationship between plaque accumulation and systemic inflammatory burden, children with ECC were stratified into low- (≤1.2), moderate- (1.21–1.80), and high- (>1.80) plaque accumulation groups based on PI tertiles (Table 3).

No statistically significant differences were observed regarding age distribution among the three PI groups (57.00 [49.00–62.00] vs. 62.50 [47.25–66.25] vs. 53.00 [46.00–61.50] months, *p* = 0.147) or sex distribution (female/male: 17/24 vs. 24/16 vs. 17/22, *p* = 0.191). Similarly, hemoglobin and hematocrit values remained comparable across the three subgroups (Hb: 13.13 ± 1.01 vs. 13.12 ± 1.16 vs. 13.15 ± 1.18 g/dL, *p* = 0.989; Ht: 39.03 ± 2.75 vs. 38.90 ± 3.51 vs. 39.99 ± 3.67%, *p* = 0.283).

In contrast, progressively increasing plaque accumulation was associated with significant elevations in several hematologic inflammatory parameters. White blood cell counts increased markedly from the low-PI group to the high-PI group (5.33 [4.94–5.95] vs. 7.38 [6.40–8.11] vs. 9.74 [8.53–11.56] × 10^3^/μL, *p* < 0.001). Similar trends were observed for neutrophil counts (2.00 [1.56–2.26] vs. 3.15 [2.52–3.84] vs. 5.00 [3.62–6.31] × 10^3^/μL, *p* < 0.001), monocyte counts (0.50 [0.40–0.56] vs. 0.55 [0.47–0.62] vs. 0.74 [0.56–0.90] × 10^3^/μL, *p* < 0.001), and platelet counts (276.00 [248.00–313.00] vs. 292.00 [249.75–329.50] vs. 352.00 [288.50–411.00] × 10^3^/μL, *p* = 0.002). Lymphocyte counts also demonstrated a modest but statistically significant increase across PI tertiles (2.76 [2.19–3.25] vs. 3.00 [2.60–3.47] vs. 2.98 [2.77–3.71] × 10^3^/μL, *p* = 0.046).

Red blood cell-related parameters showed less pronounced changes. Mean corpuscular volume demonstrated a decreasing trend with increasing plaque burden (84.80 [81.40–86.30] vs. 83.65 [80.72–85.45] vs. 82.00 [78.75–84.35] fL), although the difference did not reach statistical significance (*p* = 0.052). Conversely, RDW values increased significantly across the three groups (12.80 [12.40–13.20] vs. 12.90 [12.57–13.40] vs. 13.10 [12.65–14.20]%, *p* = 0.044). No significant differences were identified for ESR values (*p* = 0.112).

Several inflammatory hematologic indices showed strong associations with the severity of plaque accumulation. NLR values increased progressively from 0.65 (0.56–1.00) in the low-PI group to 0.91 (0.79–1.46) in the moderate-PI group and 1.54 (1.02–2.11) in the high-PI group (*p* < 0.001). Likewise, NMR values increased significantly (4.23 [3.06–5.50] vs. 5.45 [4.67–7.25] vs. 6.96 [6.00–8.26], *p* < 0.001), as did dNLR (0.54 [0.46–0.78] vs. 0.73 [0.57–1.08] vs. 1.09 [0.80–1.49], *p* < 0.001).

Among the composite inflammatory indices, AISI exhibited a marked progressive increase from 98.95 (57.37–138.28) to 150.76 (112.30–248.75) and 302.93 (214.44–625.04) (*p* < 0.001). Similar patterns were observed for SII (198.05 [113.04–276.57] vs. 267.61 [205.23–447.03] vs. 486.36 [340.87–798.35], *p* < 0.001), SIRI (0.34 [0.19–0.44] vs. 0.48 [0.43–0.85] vs. 0.91 [0.62–1.73], *p* < 0.001), and IIC (0.66 [0.60–1.10] vs. 1.00 [0.82–1.57] vs. 1.66 [1.03–2.51], *p* < 0.001). In contrast, LMR values decreased significantly with increasing plaque burden (5.95 [4.38–7.23] vs. 5.76 [4.54–6.66] vs. 4.61 [3.49–5.70], *p* = 0.011), while MCVL values also declined significantly across PI tertiles (30.72 [25.78–38.95] vs. 27.17 [23.05–31.32] vs. 27.22 [20.29–31.18], *p* = 0.039). PLR values did not differ significantly among the groups (*p* = 0.666).

Regarding inflammatory biomarkers, neither serum Interleukin 6 (IL-6) nor Interleukin 10 (IL-10) concentrations varied significantly according to plaque accumulation severity. IL-6 levels were 21.38 (18.14–22.53), 20.61 (18.24–21.82), and 20.39 (19.53–21.98) pg/mL across low-, moderate-, and high-PI groups, respectively (*p* = 0.663). Similarly, IL-10 concentrations were 39.89 (30.50–42.82), 36.08 (30.25–40.09), and 37.69 (31.23–40.27) pg/mL, respectively (*p* = 0.234).

Overall, increased plaque accumulation was associated with a progressively more pronounced systemic inflammatory profile, characterized by higher leukocyte counts and elevated composite inflammatory indices, whereas serum IL-6 and IL-10 concentrations appeared relatively stable across different plaque-burden levels. This finding suggests that hematologic inflammatory indices may be more sensitive indicators of plaque-related systemic inflammatory responses than isolated cytokine measurements in children with early childhood caries.

### 3.4. Correlation of Inflammatory and Hematologic Indices in the ECC Group

To further investigate the relationship between local oral inflammatory burden and systemic inflammatory responses, Spearman correlation analyses were performed between clinical oral indices, serum cytokines, and hematologic inflammatory markers in children with ECC (Table 4).

A strong positive correlation was observed between PI and GI scores (r = 0.811, *p* < 0.001), confirming the close relationship between plaque accumulation and gingival inflammation in children with ECC.

Increasing plaque accumulation was significantly associated with a higher systemic inflammatory burden. PI demonstrated moderate positive correlations with NLR (r = 0.536, *p* < 0.001), AISI (r = 0.648, *p* < 0.001), SII (r = 0.536, *p* < 0.001), SIRI (r = 0.654, *p* < 0.001), and IIC (r = 0.546, *p* < 0.001). Conversely, significant inverse correlations were observed between PI and LMR (r = −0.268, *p* = 0.003) and between PI and MCVL (r = −0.261, *p* = 0.004).

A similar pattern was observed for gingival inflammation. GI scores showed significant positive correlations with NLR (r = 0.411, *p* < 0.001), AISI (r = 0.478, *p* < 0.001), SII (r = 0.410, *p* < 0.001), SIRI (r = 0.480, *p* < 0.001), and IIC (r = 0.385, *p* < 0.001). In contrast, MCVL demonstrated a significant negative correlation with GI values (r = −0.301, *p* < 0.001).

Regarding inflammatory biomarkers, a strong positive association was observed between serum IL-6 and IL-10 concentrations (r = 0.804, *p* < 0.001), suggesting coordinated activation of pro-inflammatory and anti-inflammatory immune pathways. However, neither cytokine showed a significant correlation with plaque accumulation or gingival inflammation. IL-6 showed weak negative correlations with PI (r = −0.070, *p* = 0.445) and GI (r = −0.033, *p* = 0.722), while IL-10 exhibited similarly weak and non-significant associations with PI (r = −0.157, *p* = 0.087) and GI (r = −0.075, *p* = 0.414).

Overall, these findings indicate that clinical oral inflammatory severity in ECC is more closely associated with hematologic inflammatory indices than with isolated serum cytokine concentrations. The strong correlations observed between PI, GI, and multiple composite inflammatory indices suggest that plaque accumulation and gingival inflammation may contribute to measurable systemic inflammatory responses in affected children.

**Figure 2:**
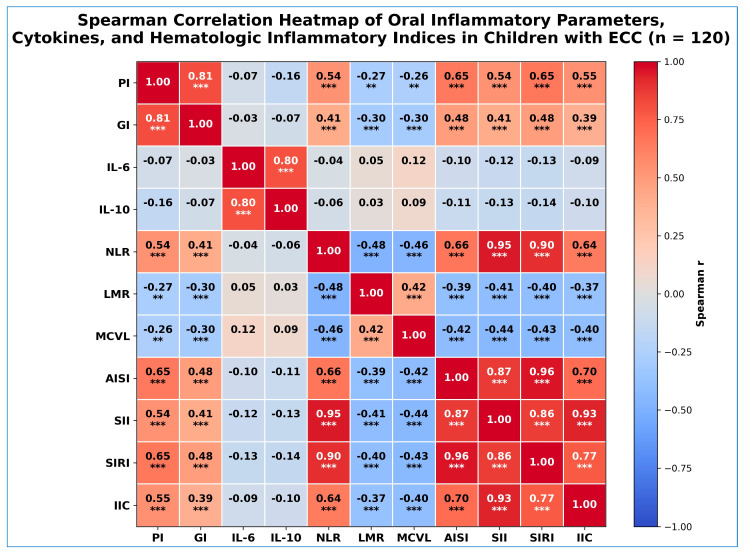
Heatmap of Correlations Between Oral Inflammatory Parameters, Cytokines, and Hematologic Indices in Children with Early Childhood Caries (ECC). Spearman correlation heatmap illustrating the relationships between local oral inflammatory parameters, serum cytokines, and hematologic inflammatory indices in children with early childhood caries (ECC; *n* = 120). Positive correlations are represented by red shades, whereas negative correlations are represented by blue shades, with color intensity corresponding to the magnitude of the correlation coefficient (r). A strong positive correlation was observed between Plaque Index (PI) and Gingival Index (GI) scores (r = 0.811, *p* < 0.001). Both PI and GI demonstrated significant positive correlations with several hematologic inflammatory indices, including neutrophil-to-lymphocyte ratio (NLR), systemic immune-inflammation index (SII), systemic inflammation response index (SIRI), aggregate index of systemic inflammation (AISI), and cumulative inflammatory index (IIC), indicating a close association between oral inflammatory severity and systemic inflammatory activation. Conversely, mean corpuscular volume-to-lymphocyte ratio (MCVL) exhibited significant inverse correlations with both PI and GI, suggesting lower MCVL values in children with greater plaque accumulation and gingival inflammation. Serum Interleukin 6 (IL-6) and Interleukin 10 (IL-10) demonstrated a strong positive intercorrelation (r = 0.804, *p* < 0.001) but showed no significant associations with PI or GI scores. Statistical significance levels are indicated as follows: ** *p* < 0.01; *** *p* < 0.001.

To facilitate visualization of the complex interactions among local oral inflammatory burden, systemic cytokine responses, and hematologic inflammatory indices, the significant correlations identified in the ECC cohort were further summarized in a heatmap (Figure 2).

### 3.5. Relationship Between Serum IL-6, IL-10, and Oral Inflammatory Severity in ECC

Given the established role of dental plaque accumulation and gingival inflammation in the pathogenesis of oral inflammatory responses, additional correlation analyses were performed to further investigate the relationship between serum cytokine levels and clinical oral inflammatory indices. Specifically, correlations between serum IL-6 and IL-10 levels and the PI and GI scores were evaluated to determine whether systemic inflammatory alterations reflect local oral biofilm burden and gingival inflammatory severity in children with ECC (Table 5).

Correlation analysis demonstrated no statistically significant associations between serum cytokine concentrations and local oral inflammatory indices within the ECC group. Serum IL-6 levels showed weak negative correlations with both PI (r = −0.070, *p* = 0.445) and GI scores (r = −0.033, *p* = 0.722). Similarly, serum IL-10 concentrations were not significantly associated with PI (r = −0.157, *p* = 0.087) or GI values (r = −0.075, *p* = 0.414), although IL-10 demonstrated a borderline inverse trend with plaque accumulation.

These findings suggest that systemic cytokine alterations observed in children with ECC may not directly parallel the severity of local plaque accumulation or gingival inflammation.

**Figure 3:**
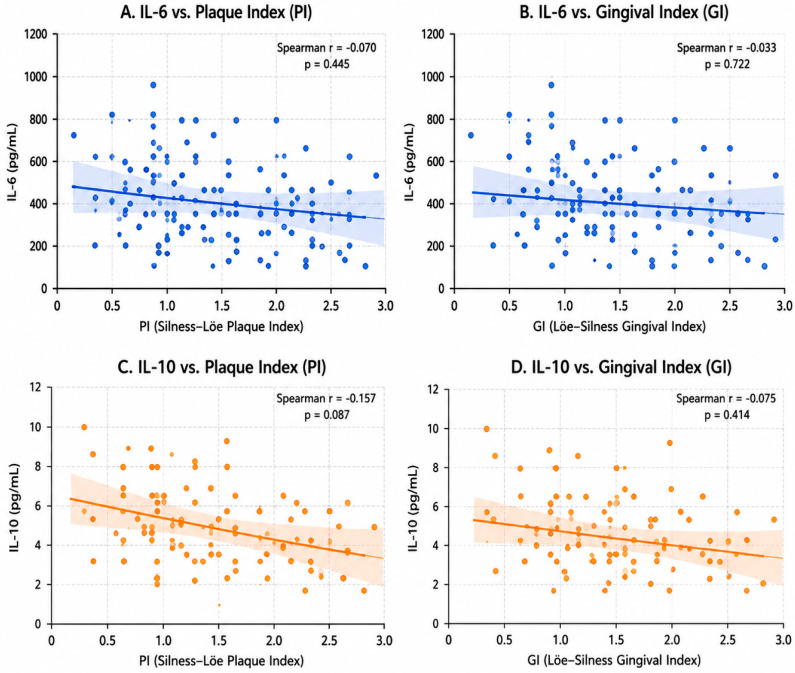
Scatter plots illustrating the relationships between serum cytokine concentrations and clinical oral inflammatory indices in children with ECC (*n* = 120). Panel (**A**) presents the association between serum Interleukin 6 (IL-6) concentrations and Plaque Index (PI) scores, while panel (**B**) illustrates the relationship between IL-6 and Gingival Index (GI). Panels (**C**,**D**) demonstrate the correlations between serum Interleukin 10 (IL-10) concentrations and PI and GI scores, respectively. Scatter points represent individual study participants. Solid lines indicate the linear regression trend, whereas shaded areas represent the 95% confidence intervals. Both the horizontal and vertical axes start at zero to facilitate standardized visualization and comparison across panels. Spearman correlation coefficients (r) and corresponding *p*-values are displayed in the upper-right corner of each graph. Weak negative correlations were identified between serum cytokine concentrations and oral inflammatory indices. Serum IL-6 demonstrated weak inverse correlations with PI (r = −0.070, *p* = 0.445) and GI (r = −0.033, *p* = 0.722), while IL-10 exhibited weak negative associations with PI (r = −0.157, *p* = 0.087) and GI (r = −0.075, *p* = 0.414). However, none of these associations reached statistical significance. These findings suggest that systemic cytokine alterations in children with ECC may not directly reflect the severity of local plaque accumulation or gingival inflammation.

Given the weak correlations observed in the previous analyses, scatter plots were additionally generated to better illustrate the direction and magnitude of the relationships between systemic cytokine profiles and local oral inflammatory parameters. Figure 3 illustrates the distribution patterns and regression trends between serum cytokine concentrations and clinical oral inflammatory indices in children with ECC.

### 3.6. Diagnostic Performance of IL-6 and IL-10 for ECC

ROC curve analysis was performed to evaluate the discriminative ability of serum IL-6 and IL-10 concentrations in distinguishing children with ECC from sibling controls.

Serum IL-6 demonstrated moderate discriminatory performance, with an AUC of 0.701. The optimal cutoff value, determined by the Youden index, was 19.90 pg/mL, with a sensitivity of 65.0% and a specificity of 80.0% (Table 6).

Similarly, serum IL-10 showed moderate discriminatory performance, with an AUC of 0.696. The optimal cutoff value was 33.83 pg/mL, yielding a sensitivity of 67.5% and a specificity of 71.4%.

These indicate that serum IL-6 and IL-10 have moderate discriminative power in differentiating children with ECC from caries-free sibling controls. However, given the observed AUC values, these findings should be considered exploratory and do not support the use of either cytokine as a standalone diagnostic biomarker. Further validation in larger prospective cohorts is warranted.

### 3.7. Age- and Sex-Adjusted Multivariable Logistic Regression Analysis of Variables Associated with ECC

Multivariate logistic regression analysis was performed to variables independently associated with ECC. To account for the significant age difference between the study groups and the potential influence of sex on inflammatory biomarkers, the regression model was adjusted for age and sex. The final model included serum IL-6 and IL-10 concentrations, as well as PLR and MCVL values. PLR and MCVL were selected as representative hematologic inflammatory markers to avoid multicollinearity among the highly correlated composite indices (NLR, SII, SIRI, AISI, and IIC) and to provide a more parsimonious multivariable model (Table 7).

Among the variables included in the age- and sex-adjusted multivariable logistic regression model, increased serum IL-6 concentrations remained independently associated with ECC (adjusted OR = 1.36; 95% CI, 1.10–1.68; *p* = 0.005). Conversely, higher MCVL values were independently associated with lower odds of ECC (adjusted OR = 0.92; 95% CI, 0.86–0.98; *p* = 0.010). Age was not independently associated with ECC after adjustment (adjusted OR = 1.12, 95% CI: 0.97–1.29, *p* = 0.128), whereas male sex was associated with increased odds of ECC (adjusted OR = 2.66, 95% CI: 1.07–6.60, *p* = 0.035). Neither serum IL-10 concentrations (adjusted OR = 0.99, 95% CI: 0.96–1.03, *p* = 0.702) nor PLR values (adjusted OR = 1.01, 95% CI: 0.99–1.02, *p* = 0.327) demonstrated independent predictive significance in the adjusted model.

These findings indicate that the associations between elevated serum IL-6 concentrations, lower MCVL values, and ECC remained significant after adjustment for the potential confounding effects of age and sex. In contrast, IL-10 and PLR did not independently contribute to predicting ECC, suggesting that systemic cytokine activation, particularly as reflected by IL-6, together with alterations in MCVL, may be more robust indicators of the inflammatory profile associated with ECC.

## 4. Discussion

The present study evaluated systemic inflammatory alterations in children with ECC using a sibling-controlled design and investigated the relationships between serum cytokine concentrations, hematologic inflammatory indices, and clinical oral inflammatory parameters. The principal findings demonstrated significantly higher serum IL-6 and IL-10 concentrations in children with ECC than in sibling controls, along with significant alterations in selected inflammatory hematologic indices, particularly MCVL. Furthermore, increasing plaque accumulation was associated with progressively elevated systemic inflammatory burden, reflected by higher NLR, SII, SIRI, AISI, and IIC values. Correlation analyses further demonstrated significant positive associations between plaque accumulation, gingival inflammation, and multiple hematologic inflammatory indices, whereas serum cytokine concentrations remained largely independent of local oral inflammatory severity. Finally, ROC analysis demonstrated moderate discriminatory performance for IL-6 and IL-10, whereas age- and sex-adjusted multivariable logistic regression demonstrated that elevated IL-6 concentrations remained independently associated with ECC, whereas higher MCVL values were associated with lower odds of ECC.

From a clinical perspective, ECC should be regarded as a chronic childhood disease with consequences extending beyond the affected tooth surface. Current pediatric dentistry policy describes ECC as the result of a sustained imbalance between risk and protective factors, while global estimates indicate that the condition affects a substantial proportion of preschool children. Untreated ECC may be associated with pain, eating difficulties, sleep disturbances, impaired oral-health-related quality of life, and increased caregiver burden. These broader consequences support the interpretation of ECC as a persistent oral disease in which cariogenic biofilm activity, plaque accumulation, and local inflammatory responses may coexist with measurable systemic biological changes. In this context, serum cytokines and CBC-derived inflammatory indices should be interpreted as adjunctive markers of host response rather than substitutes for clinical caries assessment [22,28,29].

Recent evidence increasingly supports the concept that ECC represents not only a localized infectious process but also a condition capable of inducing systemic inflammatory responses. Several studies published during the last decade have demonstrated elevated salivary and serum concentrations of pro-inflammatory cytokines, including IL-6, TNF-α, and IL-1β, in children with active carious lesions, suggesting activation of host immune-inflammatory pathways in response to cariogenic biofilm activity [10,30,31]. Consistent with these observations, our results demonstrated significantly higher serum IL-6 concentrations in children with ECC, supporting the hypothesis that chronic oral inflammatory stimulation may contribute to low-grade systemic immune activation. Recent evidence suggests that IL-6 may serve as a potential biomarker of ECC activity, with higher cytokine levels being observed in children with increasing caries burden [32,33].

Interestingly, IL-10 concentrations were also significantly elevated in the ECC group. Although IL-10 is classically regarded as an anti-inflammatory cytokine, elevated circulating levels in chronic inflammatory conditions may reflect a compensatory immunoregulatory response aimed at limiting excessive tissue damage and the progression of inflammation. Similar observations have been reported in pediatric oral inflammatory diseases and severe ECC cohorts [6,7,8,9,10,11,12]. Therefore, the simultaneous elevation of both IL-6 and IL-10 observed in our study may suggest the coexistence of pro-inflammatory activation and compensatory anti-inflammatory regulation in children with active ECC.

One of the most important novel aspects of the present study is the simultaneous evaluation of serum IL-6 and IL-10 concentrations, multiple composite hematologic inflammatory indices, and standardized clinical oral inflammatory parameters in a sibling-controlled ECC cohort. While previous studies in ECC have mainly focused on salivary cytokines and conventional inflammatory markers, evidence regarding the use of composite hematologic inflammatory indices remains limited. Most available data on indices such as SII, SIRI, AISI, and related inflammatory markers originate from periodontal diseases and other chronic inflammatory conditions rather than from pediatric caries populations [34,35]. Moreover, the sibling-controlled design represents an important methodological advantage because it reduces potential confounding related to environmental exposures, dietary habits, socioeconomic background, oral hygiene practices, and shared genetic susceptibility. Consequently, the observed differences are more likely to reflect disease-associated inflammatory alterations rather than external confounding influences.

A particularly important finding of our study was the progressive increase in inflammatory hematologic indices according to plaque accumulation severity. Children in the highest PI tertile had significantly higher WBC, neutrophil, monocyte, platelet, NLR, dNLR, NMR, SII, SIRI, AISI, and IIC values than those in the lowest PI tertile. Furthermore, PI demonstrated moderate-to-strong correlations with NLR (r = 0.536), SII (r = 0.536), SIRI (r = 0.654), AISI (r = 0.648), and IIC (r = 0.546), while inverse correlations were observed with LMR and MCVL. Similar relationships were identified for GI. These findings suggest that plaque accumulation and gingival inflammation may contribute to measurable systemic immune activation in children with ECC and support the concept that composite hematologic inflammatory indices may represent sensitive markers of plaque-related inflammatory burden.

Interestingly, despite significantly higher serum IL-6 and IL-10 concentrations in ECC patients than in sibling controls, neither cytokine showed significant correlations with PI or GI values. This observation suggests that systemic cytokine concentrations may reflect the presence of ECC rather than the local severity of plaque accumulation or gingival inflammation. A possible explanation is that cytokines reflect downstream systemic immune responses influenced by multiple host-related factors, whereas hematologic inflammatory indices may respond more directly to ongoing plaque-induced inflammation. The strong positive correlation observed between IL-6 and IL-10 (r = 0.804, *p* < 0.001) further suggests coordinated activation of pro-inflammatory and compensatory anti-inflammatory pathways within the systemic immune response. Previous studies have demonstrated that IL-6 concentrations may be influenced by oral hygiene status and plaque accumulation, although these associations have been inconsistent across pediatric populations [3]. Elevated IL-6 and IL-10 concentrations have also been observed in children with gingival inflammation, further supporting the relationship between oral inflammatory processes and systemic immune activation [12,36,37].

The correlation analyses provide additional support for the hypothesis that oral inflammatory burden is associated with systemic immune activation. Both PI and GI exhibited significant positive correlations with NLR, AISI, SII, SIRI, and IIC, whereas inverse correlations were identified with LMR and MCVL. Collectively, these findings indicate that worsening oral hygiene and increasing plaque accumulation are accompanied by measurable changes in systemic inflammatory profiles. Notably, the strongest associations were observed for SIRI and AISI, suggesting that composite indices that integrate neutrophil, monocyte, platelet, and lymphocyte counts may provide a more comprehensive reflection of the plaque-related inflammatory burden than individual hematologic parameters alone.

An additional strength of the study is the evaluation of the discriminatory performance of serum cytokines. ROC analysis demonstrated moderate discriminatory ability for both IL-6 (AUC = 0.701) and IL-10 (AUC = 0.696), with optimal cut-off values of 19.90 pg/mL and 33.83 pg/mL, respectively. The corresponding sensitivities and specificities were 65.0% and 80.0% for IL-6 and 67.5% and 71.4% for IL-10. Although these findings indicate that IL-6 and IL-10 can distinguish children with ECC from caries-free sibling controls with moderate accuracy, the observed discriminatory performance should be considered exploratory and does not support their use as standalone diagnostic biomarkers. Further validation in larger prospective cohorts is required before their potential clinical applicability can be established.

Importantly, age- and sex-adjusted multivariable logistic regression analysis demonstrated that elevated serum IL-6 concentrations (adjusted OR = 1.36, 95% CI: 1.10–1.68, *p* = 0.005) and lower MCVL values (adjusted OR = 0.92, 95% CI: 0.86–0.98, *p* = 0.010) remained independently associated with ECC, whereas neither IL-10 nor PLR retained statistical significance after adjustment. These findings indicate that the associations between IL-6, MCVL, and ECC are robust and are not solely explained by differences in age or sex between the study groups, supporting the role of systemic cytokine activation and hematologic inflammatory alterations in the inflammatory profile associated with ECC.

Beyond the observed biomarker associations, the present findings may have broader clinical implications for the overall health of children with ECC. Persistent exposure to cariogenic biofilms and chronic low-grade inflammatory stimulation may extend beyond the oral cavity, contributing to systemic immune activation reflected by elevated circulating inflammatory mediators, particularly IL-6. Although the inflammatory burden associated with ECC is substantially lower than that observed in chronic systemic inflammatory diseases, repeated immune activation during early childhood may influence immune maturation and contribute to a persistent low-grade inflammatory state. Although these systemic outcomes were not directly evaluated in the present study, the observed systemic inflammatory alterations provide additional biological support for the previously reported associations between untreated ECC, impaired nutritional status, growth disturbances, sleep disruption, and reduced quality of life. While these systemic consequences were not directly evaluated in the present study, the observed cytokine and hematologic alterations support the concept that ECC should be considered not only a localized oral disease but also a condition capable of eliciting measurable systemic immune responses. These observations further emphasize the importance of early diagnosis, preventive strategies, and timely management of ECC to reduce chronic inflammatory stimulation during a critical period of childhood development. Nevertheless, prospective longitudinal studies are required to determine whether successful treatment of ECC is accompanied by normalization of systemic inflammatory biomarkers and improved general health outcomes.

Although MCVL has been investigated as a novel hematologic inflammatory index in inflammatory and oncologic conditions, data regarding its use in pediatric dentistry remain limited. MCVL integrates erythrocyte size and lymphocyte count, thereby combining information on erythrocyte characteristics and immune cell distribution. In the present study, lower MCVL values in children with ECC may reflect subtle shifts in lymphocyte-related inflammatory activity, nutritional status, or erythrocyte parameters associated with chronic oral disease. However, this interpretation should be approached with caution, as MCVL is a derived index and its clinical meaning in pediatric oral inflammatory conditions has not been fully validated. Future studies should investigate whether MCVL remains associated with ECC after adjustment for nutritional markers, iron status, and broader inflammatory profiles [24,25,26,27].

From a translational perspective, the present findings further support the potential value of routinely available hematologic inflammatory indices for characterizing the systemic inflammatory profile associated with ECC. The observed associations between plaque accumulation severity and multiple hematologic inflammatory indices suggest that these readily available CBC-derived parameters reflect systemic immune activation accompanying oral inflammatory burden. Because indices such as NLR, SII, SIRI, AISI, IIC, and MCVL can be calculated from routine CBC results without additional laboratory costs, they may represent promising candidate biomarkers for exploratory risk stratification and for monitoring systemic inflammatory responses in future research settings. Furthermore, the persistence of the associations between IL-6 and MCVL, and ECC after adjustment for age and sex suggests that these biomarkers capture biological alterations beyond demographic differences. However, given the observational design of the present study and the moderate discriminatory performance observed in ROC analysis, these biomarkers should currently be regarded as exploratory indicators rather than clinically applicable diagnostic tools. Future prospective multicenter studies are needed to validate their reproducibility, determine their incremental value beyond conventional clinical assessment, and clarify their potential role in personalized risk assessment and longitudinal monitoring of children with ECC.

Several limitations of the study should be acknowledged. First, the cross-sectional case–control design precludes causal inferences and limits the ability to establish a temporal relationship between ECC and systemic inflammatory alterations. Second, although all participants were within the early-childhood age range, the ECC and sibling control groups differed significantly in age. To address this potential source of confounding, an age- and sex-adjusted multivariable logistic regression analysis was performed, and the principal associations between IL-6, MCVL, and ECC remained statistically significant after adjustment. Nevertheless, residual confounding by age and sex cannot be completely ruled out, and future studies should include larger age-matched sibling-control cohorts. Third, cytokine assessment was limited to IL-6 and IL-10, while other important inflammatory mediators, including TNF-α, IL-1β, IL-17, and hs-CRP, were not evaluated. In addition, microbiological characterization of the cariogenic biofilm was not performed, preventing direct assessment of the relationship between microbial burden and systemic inflammatory responses. Another limitation concerns the age imbalance between ECC patients and sibling controls. Although all participants were within the early-childhood age range, the control group was older than the ECC group, and age-related differences may influence oral disease status, hematologic parameters, and cytokine concentrations. Therefore, residual confounding related to age and sex cannot be fully excluded. Future studies should include larger age-matched sibling-control cohorts and age- and sex-adjusted regression models to confirm the independent value of IL-6 and MCVL. Another limitation is that quantitative measures of caries severity, such as dmft/dmfs scores or the number of cavitated lesions, were not recorded. Consequently, the present study could not evaluate dose-dependent associations between caries burden and systemic inflammatory biomarkers. Future studies incorporating standardized caries severity indices are warranted to determine whether increasing disease severity is accompanied by progressive systemic immune activation. Additionally, multiple hematologic variables and derived inflammatory indices were analyzed without formal correction for multiple comparisons; therefore, borderline findings should be interpreted cautiously and require confirmation in larger cohorts. Another methodological limitation relates to cytokine quantification. Although all serum samples were analyzed under identical laboratory conditions using standardized commercial ELISA kits, each sample was measured only once, and laboratory-specific intra- and inter-assay precision was not independently evaluated. Consequently, measurement variability cannot be completely excluded. However, the assays used were validated commercial kits with intra- and inter-assay performance characteristics provided by the manufacturer. Future studies should incorporate duplicate or triplicate measurements together with laboratory-specific validation of assay precision to further strengthen the reproducibility of cytokine measurements. Furthermore, although all samples were processed according to standardized institutional laboratory procedures, fasting status, exact blood collection time, and the interval between blood collection and processing were not prospectively standardized or systematically recorded. Therefore, some degree of pre-analytical variability cannot be excluded. Finally, longitudinal follow-up after dental treatment was unavailable; therefore, the potential normalization of inflammatory biomarkers following successful ECC management could not be investigated.

Despite these limitations, the present study provides novel evidence linking ECC, plaque accumulation severity, systemic cytokine alterations, and hematologic inflammatory indices in a sibling-controlled pediatric cohort. The findings suggest that the plaque-related inflammatory burden is better reflected by composite hematologic inflammatory indices than by isolated serum cytokine concentrations. Future longitudinal multicenter studies incorporating larger pediatric cohorts, comprehensive inflammatory profiling, and post-treatment follow-up are warranted to further clarify the role of systemic inflammatory biomarkers in the pathophysiology, risk stratification, and clinical monitoring of early childhood caries.

## 5. Conclusions

ECC is associated with significant systemic inflammatory alterations, reflected by elevated serum IL-6 and IL-10 concentrations and changes in selected hematologic inflammatory indices. Increasing plaque accumulation was associated with a progressively higher inflammatory burden, with significant associations among the PI, GI, and several composite hematologic markers, including NLR, SII, SIRI, AISI, and IIC. While IL-6 and IL-10 differentiated children with ECC from caries-free sibling controls, they were not significantly associated with plaque accumulation or the severity of gingival inflammation. In contrast, hematologic inflammatory indices appeared to more accurately reflect local oral inflammatory burden. Moreover, elevated IL-6 concentrations remained independently associated with ECC, whereas increasing MCVL values were associated with reduced odds of ECC. Overall, these findings suggest that ECC was associated with systemic inflammatory alterations and that composite hematologic inflammatory indices may provide complementary information regarding plaque-related inflammatory burden. Further longitudinal studies are needed to validate their clinical utility and clarify their role in the biological mechanisms underlying ECC.

## Figures and Tables

**Table 1:** Comparative Analysis Between ECC and Sibling Controls.

Variable	ECC Group (*n* = 120)	Control (*n* = 35)	*p*-Value
Age (months), Median (IQR)	57 (47–64)	67 (65–69)	<0.0001
Sex (Male/Female), *n*	62/58	13/22	0.130
Hb (g/dL), Mean ± SD	13.13 ± 1.11	12.84 ± 0.93	0.118
Ht (%), Mean ± SD	39.30 ± 3.33	38.56 ± 2.27	0.134
WBC (×10^3^/μL), Median (IQR)	7.38 (5.84–8.97)	6.38 (5.14–6.88)	<0.001
NEU (×10^3^/μL), Median (IQR)	2.91 (2.20–4.53)	2.35 (1.88–3.20)	0.044
LYM (×10^3^/μL), Median (IQR)	2.94 (2.55–3.46)	2.44 (2.15–3.14)	0.020
MON (×10^3^/μL), Median (IQR)	0.55 (0.47–0.70)	0.51 (0.38–0.64)	0.186
PLT (×10^3^/μL), Median (IQR)	294.5 (250.0–352.0)	292.0 (276.0–321.5)	0.603
MCV (fL), Median (IQR)	83.05 (79.88–85.40)	83.50 (81.50–85.55)	0.132
RDW (%), Median (IQR)	12.90 (12.50–13.40)	12.80 (12.35–14.10)	0.745
ESR (mm/h), Median (IQR)	9.0 (6.0–10.0)	6.0 (5.0–8.0)	0.005
Hematologic indices			
NLR, Median (IQR)	0.98 (0.65–1.55)	0.75 (0.61–1.21)	0.350
LMR, Median (IQR)	5.41 (4.06–6.76)	5.41 (4.84–6.32)	0.834
PLR, Median (IQR)	99.17 (80.09–125.75)	128.84 (89.92–132.48)	0.0667 #
NMR, Median (IQR)	5.50 (4.36–7.12)	4.68 (3.59–6.50)	0.183
dNLR, Median (IQR)	0.75 (0.50–1.14)	0.59 (0.48–0.82)	0.073
AISI, Median (IQR)	174.46 (98.95–291.41)	135.64 (85.54–200.95)	0.123
SII, Median (IQR)	278.56 (199.96–497.15)	274.37 (189.26–398.02)	0.396
SIRI, Median (IQR)	0.50 (0.34–0.90)	0.44 (0.32–0.51)	0.105
Novel hematologic indices			
MCVL, Median (IQR)	28.51 (22.32–32.48)	34.10 (26.51–38.62)	0.0109
IIC, Median (IQR)	1.01 (0.67–1.70)	0.78 (0.74–1.43)	0.419
Biomarkers			
IL-6 (pg/mL), Median (IQR)	20.70 (18.77–22.15)	18.29 (17.17–19.54)	0.0003
IL-10 (pg/mL), Median (IQR)	36.58 (30.49–41.33)	30.29 (25.78–35.63)	0.0004

Data are presented as mean ± SD for normally distributed variables and median (IQR) for non-normally distributed variables. Group comparisons were performed using the independent-samples *t*-test or the Mann–Whitney U test, as appropriate. Categorical variables were compared using the chi-square test or Fisher’s exact test; #: borderline difference.

**Table 2:** Comparative Analysis Between Females and Males in the ECC Group.

Variable	Females (*n* = 58)	Males (*n* = 62)	*p*-Value
Age (months) Median (IQR)	60.50 (47.50–65.00)	55.50 (47.00–63.25)	0.365
Hb (g/dL) Mean ± SD	12.65 ± 1.06	12.77 ± 1.15	0.548
Ht (%) Mean ± SD	38.20 ± 2.54	38.51 ± 2.89	0.536
WBC (×10^3^/μL) Median (IQR)	7.24 (5.80–8.92)	7.41 (5.91–9.01)	0.864
NEU (×10^3^/μL) Median (IQR)	2.88 (2.14–4.41)	2.95 (2.28–4.61)	0.771
LYM (×10^3^/μL) Median (IQR)	2.99 (2.59–3.52)	2.91 (2.50–3.44)	0.618
MON (×10^3^/μL) Median (IQR)	0.54 (0.45–0.69)	0.56 (0.48–0.71)	0.490
PLT (×10^3^/μL) Median (IQR)	298.00 (248.00–360.00)	291.00 (244.00–349.00)	0.681
MCV (fL) Median (IQR)	81.40 (78.10–84.40)	81.70 (78.40–84.90)	0.790
RDW (%) Median (IQR)	13.20 (12.70–13.70)	13.00 (12.60–13.50)	0.329
ESR (mm/h) Median (IQR)	9.00 (6.00–14.00)	9.00 (6.00–13.00)	0.941
Hematologic indices			
NLR, Median (IQR)	0.94 (0.72–1.50)	0.98 (0.74–1.57)	0.664
LMR, Median (IQR)	5.24 (4.18–6.35)	5.09 (4.09–6.18)	0.747
PLR, Median (IQR)	99.34 (77.62–126.48)	96.85 (75.11–122.33)	0.905
NMR, Median (IQR)	5.28 (3.81–7.22)	5.42 (3.95–7.55)	0.824
dNLR, Median (IQR)	0.63 (0.47–0.95)	0.65 (0.49–1.00)	0.631
AISI, Median (IQR)	170.35 (110.44–292.15)	165.12 (106.91–284.84)	0.324
SII, Median (IQR)	292.60 (192.11–485.74)	285.44 (186.52–471.60)	0.575
SIRI, Median (IQR)	0.51 (0.33–0.86)	0.53 (0.35–0.89)	0.164
New Hematologic indices			
MCVL, Median (IQR)	28.61 (23.18–34.20)	27.98 (22.66–33.21)	0.249
IIC, Median (IQR)	0.88 (0.73–1.07)	0.86 (0.71–1.03)	0.333
Biomarkers			
IL-6 (pg/mL) Median (IQR)	22.81 (17.66–28.95)	22.03 (17.11–28.02)	0.679
IL-10 (pg/mL) Median (IQR)	42.11 (34.25–52.36)	39.80 (32.90–49.64)	0.048

Data are presented as mean ± SD for normally distributed variables and median (IQR) for non-normally distributed variables. Comparisons among plaque index tertiles were performed using one-way ANOVA with Tukey’s post hoc test for normally distributed variables or the Kruskal–Wallis test followed by Dunn’s multiple-comparison test for non-normally distributed variables.

**Table 3:** Comparative Analysis According to Plaque Index Severity in Children with ECC.

Variable	Low PI (*n* = 41)	Moderate PI (*n* = 40)	High PI (*n* = 39)	*p*-Value
Age (months)	57.00 (49.00–62.00)	62.50 (47.25–66.25)	53.00 (46.00–61.50)	0.147
Sex (Female/Male)	17/24	24/16	17/22	0.191
Hb (g/dL)	13.13 ± 1.01	13.12 ± 1.16	13.15 ± 1.18	0.989
Ht (%)	39.03 ± 2.75	38.90 ± 3.51	39.99 ± 3.67	0.283
WBC (×10^3^/μL)	5.33 (4.94–5.95)	7.38 (6.40–8.11)	9.74 (8.53–11.56)	<0.001
NEU (×10^3^/μL)	2.00 (1.56–2.26)	3.15 (2.52–3.84)	5.00 (3.62–6.31)	<0.001
LYM (×10^3^/μL)	2.76 (2.19–3.25)	3.00 (2.60–3.47)	2.98 (2.77–3.71)	0.046
MON (×10^3^/μL)	0.50 (0.40–0.56)	0.55 (0.47–0.62)	0.74 (0.56–0.90)	<0.001
PLT (×10^3^/μL)	276.00 (248.00–313.00)	292.00 (249.75–329.50)	352.00 (288.50–411.00)	0.002
MCV (fL)	84.80 (81.40–86.30)	83.65 (80.72–85.45)	82.00 (78.75–84.35)	0.052
RDW (%)	12.80 (12.40–13.20)	12.90 (12.57–13.40)	13.10 (12.65–14.20)	0.044
ESR (mm/h)	5.00 (5.00–6.00)	6.00 (5.00–9.25)	6.00 (5.00–10.00)	0.112
Hematologic indices				
NLR	0.65 (0.56–1.00)	0.91 (0.79–1.46)	1.54 (1.02–2.11)	<0.001
LMR	5.95 (4.38–7.23)	5.76 (4.54–6.66)	4.61 (3.49–5.70)	0.011
PLR	100.61 (72.46–123.21)	93.30 (80.09–123.12)	101.84 (82.15–140.36)	0.666
NMR	4.23 (3.06–5.50)	5.45 (4.67–7.25)	6.96 (6.00–8.26)	<0.001
dNLR	0.54 (0.46–0.78)	0.73 (0.57–1.08)	1.09 (0.80–1.49)	<0.001
AISI	98.95 (57.37–138.28)	150.76 (112.30–248.75)	302.93 (214.44–625.04)	<0.001
SII	198.05 (113.04–276.57)	267.61 (205.23–447.03)	486.36 (340.87–798.35)	<0.001
SIRI	0.34 (0.19–0.44)	0.48 (0.43–0.85)	0.91 (0.62–1.73)	<0.001
New Hematologic indices				
MCVL	30.72 (25.78–38.95)	27.17 (23.05–31.32)	27.22 (20.29–31.18)	0.039
IIC	0.66 (0.60–1.10)	1.00 (0.82–1.57)	1.66 (1.03–2.51)	<0.001
Biomarkers				
IL-6 (pg/mL)	21.38 (18.14–22.53)	20.61 (18.24–21.82)	20.39 (19.53–21.98)	0.663
IL-10 (pg/mL)	39.89 (30.50–42.82)	36.08 (30.25–40.09)	37.69 (31.23–40.27)	0.234

Data are presented as mean ± SD for normally distributed variables and median (IQR) for non-normally distributed variables. Comparisons among plaque index tertiles were performed using one-way ANOVA with Tukey’s post hoc test for normally distributed variables or the Kruskal–Wallis test followed by Dunn’s multiple-comparison test for non-normally distributed variables.

**Table 4:** Spearman Correlation Analysis Between Oral Inflammatory Parameters, Cytokines, and Hematologic Indices in Children with ECC.

Variables	r	*p*-Value
PI vs. GI	0.811	<0.001
PI vs. NLR	0.536	<0.001
PI vs. LMR	−0.268	0.003
PI vs. AISI	0.648	<0.001
PI vs. SII	0.536	<0.001
PI vs. SIRI	0.654	<0.001
PI vs. MCVL	−0.261	0.004
PI vs. IIC	0.546	<0.001
GI vs. NLR	0.411	<0.001
GI vs. AISI	0.478	<0.001
GI vs. SII	0.410	<0.001
GI vs. SIRI	0.480	<0.001
GI vs. MCVL	−0.301	<0.001
GI vs. IIC	0.385	<0.001
IL-6 vs. IL-10	0.804	<0.001
IL-6 vs. PI	−0.070	0.445
IL-6 vs. GI	−0.033	0.722
IL-10 vs. PI	−0.157	0.087
IL-10 vs. GI	−0.075	0.414

**Table 5:** Correlations Between Serum Cytokines and Clinical Oral Inflammatory Indices in the ECC Group.

Variables	Spearman r	*p*-Value
IL-6 vs. PI	−0.070	0.445
IL-6 vs. GI	−0.033	0.722
IL-10 vs. PI	−0.157	0.087
IL-10 vs. GI	−0.075	0.414

**Table 6:** ROC Analysis of Serum Cytokines for Discrimination Between Caries and Control Groups.

Biomarker	AUC	Optimal Cutoff (Youden)	Sensitivity (%)	Specificity (%)
IL-6	0.701	19.90 pg/mL	65.0	80.0
IL-10	0.696	33.83 pg/mL	67.5	71.4

**Table 7:** Age- and Sex-Adjusted Multivariate Logistic Regression Analysis of Factors Independently Associated with ECC.

Variable	OR	95% CI	*p*-Value
Age	1.12	0.97–1.29	0.128
Male sex	2.66	1.07–6.60	0.035
IL-6	1.36	1.10–1.68	0.005
IL-10	0.99	0.96–1.03	0.702
PLR	1.01	0.99–1.02	0.327
MCVL	0.92	0.86–0.98	0.010

OR, odds ratio; CI, confidence interval. Dependent variable: ECC status (ECC = 1; caries-free sibling controls = 0).

## Data Availability

The authors declare that the data of this research are available from the corresponding authors upon reasonable request.
